# Optimal experimental design and estimation for q‐space trajectory imaging

**DOI:** 10.1002/hbm.26175

**Published:** 2022-12-23

**Authors:** Jan Morez, Filip Szczepankiewicz, Arnold J. den Dekker, Floris Vanhevel, Jan Sijbers, Ben Jeurissen

**Affiliations:** ^1^ imec‐Vision Lab, Department of Physics University of Antwerp Antwerp Belgium; ^2^ μNEURO Research Centre of Excellence University of Antwerp Antwerp Belgium; ^3^ Department of Diagnostic Radiology, Clinical Sciences Lund Lund University Lund Sweden; ^4^ Department of Radiology University Hospital Antwerp Antwerp Belgium; ^5^ Lab for Equilibrium Investigations and Aerospace, Department of Physics University of Antwerp Antwerp Belgium

**Keywords:** acquisition, diffusion magnetic resonance imaging, optimal experimental design, parameter estimation, q‐space trajectory imaging, tensor‐valued diffusion encoding

## Abstract

Tensor‐valued diffusion encoding facilitates data analysis by q‐space trajectory imaging. By modeling the diffusion signal of heterogeneous tissues with a diffusion tensor distribution (DTD) and modulating the encoding tensor shape, this novel approach allows disentangling variations in diffusivity from microscopic anisotropy, orientation dispersion, and mixtures of multiple isotropic diffusivities. To facilitate the estimation of the DTD parameters, a parsimonious acquisition scheme coupled with an accurate and precise estimation of the DTD is needed. In this work, we create two precision‐optimized acquisition schemes: one that maximizes the precision of the raw DTD parameters, and another that maximizes the precision of the scalar measures derived from the DTD. The improved precision of these schemes compared to a naïve sampling scheme is demonstrated in both simulations and real data. Furthermore, we show that the weighted linear least squares (WLLS) estimator that uses the squared reciprocal of the noisy signal as weights can be biased, whereas the iteratively WLLS estimator with the squared reciprocal of the predicted signal as weights outperforms the conventional unweighted linear LS and nonlinear LS estimators in terms of accuracy and precision. Finally, we show that the use of appropriate constraints can considerably increase the precision of the estimator with only a limited decrease in accuracy.

## Graphical abstract

In this work, we propose a parsimonious acquisition scheme that maximizes the precision of the scalar metrics, which can improve the precision of the resulting scalar metrics by a factor of up to 3.75 compared to a naive acquisition scheme. In addition, we propose the use of an iteratively reweighted linear least squares estimator in combination with QTI‐specific constraints that results in high‐quality parameter maps, even when the q‐space is sparsely sampled.

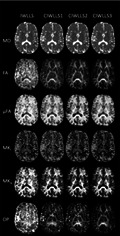

## 1 INTRODUCTION

Diffusion‐weighted magnetic resonance imaging (DW‐MRI) provides a unique look into the microstructure of the human brain, in vivo and noninvasively. This is achieved by sensitizing the signal to the random motion of water molecules that permeate biological tissues. In isotropic tissues such as gray matter (GM) and cerebrospinal fluid (CSF), the apparent diffusivity of water molecules can be characterized by a scalar quantity. However, in anisotropic tissues such as white matter (WM), the apparent diffusivity will depend on the direction along which it is probed, and a tensor‐valued quantity known as the diffusion tensor is more appropriate (Chenevert et al., 1990; Moseley et al., 1990).

Conventional DW‐MRI methods such as diffusion tensor imaging (DTI) estimate the voxel‐averaged diffusion tensor by applying diffusion weighting along multiple spatial directions (Basser et al., 1994). The strength of the diffusion weighting is typically described by a scalar quantity called the *b*‐value (Le Bihan et al., 1986), and the direction along which the diffusion weighting is applied can be described by a unit vector. Within the context of DTI, a single DW measurement thus is characterized by a b‐vector, or equivalently a b‐tensor of rank one (Basser et al., 1994).

Alternatively, q‐space trajectory imaging (QTI) enables diffusion weighting using higher‐rank b‐tensors (Westin et al., 2014, 2016). By assuming that heterogeneous tissues can be modeled by a diffusion tensor distribution (DTD), these b‐tensors can be used to disentangle microscopic anisotropy (cell shape), orientation dispersion (cell orientation), and heterogeneity of isotropic diffusivity (cell size; Szczepankiewicz et al., 2015, 2016). Compared to conventional DTI, QTI can thus provide more specific tissue metrics to assess tissue anisotropy or to characterize cancers (Andersen et al., 2020; Kamiya et al., 2020; Lampinen, Zampeli, et al., 2020b; Langbein et al., 2021; Li et al., 2021; Nilsson et al., 2020, 2021; Szczepankiewicz et al., 2016; Yang et al., 2018).

Studies that use these novel QTI‐derived tissue metrics as a biomarker could greatly benefit from a precision‐maximizing QTI sampling scheme. Currently, it is not immediately obvious how the *b* values and b‐tensor shapes of a given number of DW samples should be distributed to achieve maximal precision in the estimation of QTI‐derived tissue metrics. (Coelho et al., 2019) previously explored optimal experiment design for QTI using only simulations, but they did not consider the impact on the final QTI‐derived tissue metrics during their optimization. A common choice is to use a combination of the three major encodings (i.e., linear, planar, and spherical tensor encoding) at approximately regularly spaced *b* values (Nilsson et al., 2020; Szczepankiewicz, Hoge, & Westin, 2019). However, these *b* values might not guarantee the highest precision, as was demonstrated for diffusion kurtosis imaging (DKI) by Poot et al. (2010).

Not only the sampling strategy but also the choice of the estimator can considerably impact the bias and precision with which the tissue parameters are estimated. As the DTD model can be linearized using the natural logarithm, the linear least squares (LLS) estimator is a common choice due to its ease of implementation and the closed‐form solution it provides. However, it is known that the variance of the log‐transformed DW signal is no longer constant (Basser et al., 1994). As such, the LLS estimator, which assumes a constant variance across all DW samples, will have suboptimal precision. To combat this, a weighted linear least squares (WLLS) estimator using the squared reciprocals of the noisy signal as weights will provide improved precision (Basser et al., 1994). However, Veraart et al. (2013) showed for DKI that using these particular weights for a WLLS estimator may introduce a bias. An iteratively weighted linear least squares (IWLLS) estimator with its weights based on the squared inverse of the predicted signal is expected to provide more accurate and precise parameter estimates compared to WLLS and even nonlinear least squares (NLS; Veraart et al., 2013). Moreover, imposing certain constraints that follow from the physics of diffusion has the potential to dramatically improve the precision of DTD parameters (Basser & Pajevic, 2007; Herberthson et al., 2021; Tabesh et al., 2011; Veraart et al., 2011).

In this work, we propose two optimized parsimonious sampling schemes and compare them to a naive sampling scheme in terms of attainable precision. In addition, we evaluate the precision and accuracy of various linear and nonlinear DTD parameter estimators, as well as various constrained iteratively weighted linear DTD parameter estimators.

## 2 THEORY

In this section, we provide a short overview of the DTD and its parametrization in the QTI framework, followed by the definition of the forward model based on the cumulant expansion. Next, we introduce the Cramér‐Rao lower bound and we define the optimality criteria used to obtain optimal sampling schemes. Lastly, we introduce the various estimators and constraints evaluated in this work.

### 2.1 The diffusion tensor distribution

The QTI framework accounts for the heterogeneity of biological tissue by modeling the fully symmetric second‐order diffusion tensor D as a random variable having a tensor‐variate distribution $P\left(D\right)$ (Basser & Pajevic, 2003, 2007; Jian et al., 2007). Under this assumption, the DW signal probed with a b‐tensor B becomes a linear superposition of diffusion tensors D, weighted with $P\left(D\right)$:

(1) $$S=S_{0}\int P\left(D\right)\exp\left(-B:D\right)dD=S_{0}\left\langle \exp\left(-B:D\right)\right\rangle ,$$

where B:D is the inner tensor product, $S_{0}$ is the signal without diffusion weighting, and $\left\langle .\right\rangle$ is the expectation value operator. Several statistical metrics can be calculated for the DTD $P\left(D\right)$, such as its expectation value (Topgaard & Söderman, 2002; Westin et al., 2016):

(2) $$\begin{array}{c} \left\langle D\right\rangle =\int DP\left(D\right)dD, \end{array}$$

as well as the fourth‐order covariance tensor ℂ (Westin et al., 2014):

(3) $$\begin{array}{c} \mathbb{C}=\left\langle D^{⨂2}\right\rangle -{\left\langle D\right\rangle }^{⨂2}, \end{array}$$

where $D^{⨂2}$ is the outer tensor product of D with itself (Basser & Pajevic, 2007). The two‐term cumulant expansion of Equation (1) is then given by Westin et al. (2014, 2016):

(4) $$\begin{array}{c} S=S_{0}\;\exp\left(-B:\left\langle D\right\rangle +1/2\;B^{⨂2}:\mathbb{C}\right). \end{array}$$

For computational convenience, we follow the tensor formalism by Westin et al. (2016) and Nilsson et al. (2018), where the second‐ and fourth‐order tensors are stored as vectors. For example, the (fully symmetric) second‐order diffusion tensor D can be represented as a 6×1 column vector:

(5) $$\begin{array}{c} d={\left(d_{\mathrm{xx}}\;d_{\mathrm{yy}}\;d_{\mathrm{zz}}\;\sqrt{2}d_{\mathrm{yz}}\;\sqrt{2}d_{\mathrm{xz}}\;\sqrt{2}d_{\mathrm{xy}}\right)}^{T}, \end{array}$$

where the $\sqrt{2}$ factors are normalization factors. The fourth‐order diffusion covariance tensor ℂ with major and minor symmetry (${\mathbb{C}}_{\mathrm{ij},\mathrm{kl}}={\mathbb{C}}_{\mathrm{kl},\mathrm{ij}}$ and ${\mathbb{C}}_{\mathrm{ij},\mathrm{kl}}={\mathbb{C}}_{\mathrm{ji},\mathrm{lk}}$) can be represented as a 21×1 column vector:

(6) $$\begin{array}{c} c={\left(c_{\mathrm{xx},\mathrm{xx}}\;c_{\mathrm{xx},\mathrm{yy}}\ldots \sqrt{2}c_{\mathrm{xz},\mathrm{yz}}\right)}^{T}. \end{array}$$

### 2.2 Forward signal model

Consider the N×1 vector $s={\left(S^{\left(1\right)}\;\;S^{\left(2\right)}\ldots S^{\left(N\right)}\right)}^{T}$ representing the N diffusion‐weighted measurements, and the 28×1 parameter vector θ:

(7) $$\begin{array}{c} \theta ={\left(\log\;S_{0}\;d\;c\right)}^{T}={\left(\log\;S_{0}\;d_{\mathrm{xx}}\;d_{\mathrm{yy}}\ldots \sqrt{2}d_{\mathrm{xz}}\;\sqrt{2}d_{\mathrm{xy}}\ldots c_{\mathrm{xx},\mathrm{xx}}\;c_{\mathrm{xx},\mathrm{yy}}\ldots \sqrt{2}c_{\mathrm{xz},\mathrm{yz}}\right)}^{T}, \end{array}$$

where $d_{\mathrm{ij}}$ corresponds to the six independent parameters of $\left\langle D\right\rangle$ and $c_{\mathrm{ij},\mathrm{kl}}$ to the 21 independent parameters of ℂ (with the index pairs ij and $\mathrm{kl}\in \left\{\mathrm{xx},\mathrm{yy},\mathrm{zz},\mathrm{xy},\mathrm{xz},\mathrm{yz}\right\})$. The forward signal model can then be expressed compactly as:

(8) $$\begin{array}{c} s\left(\theta \right)=\exp\left(A\theta \right), \end{array}$$

with A the N×28 design matrix (Nilsson et al., 2018):

(9) $$\begin{array}{c} A=\left(\begin{array}{ccccccc} 1 & -b_{\mathrm{xx}}^{\left(1\right)} & \cdots & -\sqrt{2}b_{\mathrm{yx}}^{\left(1\right)} & {1/2\;b}_{\mathrm{xx},\mathrm{xx}}^{\left(1\right)} & \ldots & \sqrt{8}/2\;b_{\mathrm{xz},\mathrm{yz}}^{\left(1\right)}\\ 1 & {-b}_{\mathrm{xx}}^{\left(2\right)} & \cdots & -\sqrt{2}b_{\mathrm{yx}}^{\left(2\right)} & {1/2\;b}_{\mathrm{xx},\mathrm{xx}}^{\left(2\right)} & \ldots & {\sqrt{8}/2\;b}_{\mathrm{xz},\mathrm{yz}}^{\left(2\right)}\\ \vdots & \vdots & \vdots & \vdots & \vdots & \vdots & \vdots \\ 1 & -b_{\mathrm{xx}}^{\left(N\right)} & \cdots & -\sqrt{2}b_{\mathrm{yx}}^{\left(N\right)} & {1/2\;b}_{\mathrm{xx},\mathrm{xx}}^{\left(N\right)} & \ldots & {\sqrt{8}/2\;b}_{\mathrm{xz},\mathrm{yz}}^{\left(N\right)} \end{array}\right), \end{array}$$

where $b_{\mathrm{ij}}$ are the components of the second‐order tensor B and $b_{\mathrm{ij},\mathrm{kl}}$ the components of the fourth‐order tensor $B^{⨂2}$ (in Voight notation). The complete expression of Equation (9) can be retrieved from the appendix of Westin et al. (2016).

While any b‐tensor shape could in principle be used to sample the DW data, in this work we will only consider axisymmetric b‐tensors, which have the following diagonal form in their principal axis system (PAS):

(10) $$\begin{array}{c} \;B_{\mathrm{PAS}}=\left(\begin{array}{ccc} b_{⊥} & 0 & 0\\ 0 & b_{⊥} & 0\\ 0 & 0 & b_{∥} \end{array}\right), \end{array}$$

where $b_{⊥}$ and $b_{\|}$ are the radial and axial eigenvalues of the b‐tensor, respectively. The anisotropy $b_{∆}$ of the b‐tensor is defined as (Eriksson et al., 2015):

(11) $$\begin{array}{c} b_{\Delta }=\frac{b_{∥}-b_{⊥}}{b_{∥}+2b_{⊥}}, \end{array}$$

with values ranging between −0.5 (planar tensor encoding, or PTE) and 1 (linear tensor encoding, or LTE). Spherical tensor encoding (STE) corresponds with $b_{∆}=0$ and yields isotropic diffusion weighting.

A more compact representation of the experimental parameters can be achieved by defining an N×5 acquisition scheme matrix Q:

(12) $$\begin{array}{c} Q=\left(\begin{array}{ccc} G & b & b_{\Delta } \end{array}\right)=\left(\begin{array}{ccc} g_{1} & b_{1} & b_{\Delta ,1}\\ g_{2} & b_{2} & b_{\Delta ,2}\\ \vdots & \vdots & \vdots \\ g_{N} & b_{N} & b_{\Delta ,N} \end{array}\right), \end{array}$$

where G is an N×3 matrix containing the N unit row vectors $g_{i}=\left(g_{x}^{\left(i\right)}\;g_{y}^{\left(i\right)}\;g_{z}^{\left(i\right)}\right)$ representing the direction of the principal axis of each b‐tensor, b is an N×1 column vector containing the *b* values, and $b_{\Delta }$ is an N×1 column vector containing the b‐tensor anisotropies associated with each DW sample. As the signal model remains the same throughout this work, the design matrix A will only vary with the experimental parameters contained in Q. Consequently, we have that $A=A\left(Q\right)$, and any function that depends on A can also be considered a function of Q. This property will be used in the following section.

### 2.3 Optimal experimental design

In this section, we describe how maximally precise acquisition schemes can be obtained by minimizing several criteria based on the Cramér–Rao lower bound (CRLB). Previous work has used the CRLB to optimize diffusion acquisition settings (Alexander, 2008; Brihuega‐Moreno et al., 2003; Caan et al., 2010; Coelho et al., 2019; Jalnefjord et al., 2019; Lampinen, Szczepankiewicz, et al., 2020a; Peña‐Nogales et al., 2020; Poot et al., 2010; Slator et al., 2019; Zhang et al., 2013). The CRLB provides a lower bound for the covariance of any unbiased estimator $\hat{\theta }$ of θ, and it can be calculated by inverting the Fisher information matrix (FIM). Its diagonal elements provide a theoretical lower bound for the variance of $\hat{\theta }.$For the model described in Equation (8), assuming independent and zero mean identically Gaussian distributed noise with variance $\sigma^{2}$, the FIM takes the following form (van den Bos, 2007, p. 52):

(13) $$\begin{array}{c} I\left(\theta ,A\right)=\frac{1}{\sigma^{2}}{\left[s\left(\theta \right)A\right]}^{T}\;s\left(\theta \right)A \end{array}$$

The FIM for a set of K derived metrics $m_{i}\left(\theta \right)$ (with i=1,2,…,K) can be calculated as follows (van den Bos, 2007, p. 54):

(14) $$\begin{array}{c} J\left(\theta ,A\right)=M^{T}I{\left(\theta ,A\right)}^{-1}M, \end{array}$$

where the 28×K matrix M is defined as:

(15) $$\begin{array}{c} M=\left(\begin{array}{cccc} \frac{\partial m_{1}}{\partial \theta } & \frac{\partial m_{2}}{\partial \theta } & \cdots & \frac{\partial m_{K}}{\partial \theta } \end{array}\right).\; \end{array}$$

Several optimality criteria are available based on either Equation (13) or Equation (14). A commonly used criterion to be minimized is the product of the variances, or equivalently the determinant of the CRLB (known as D‐optimal design; Pukelsheim, 2006). The objective function then takes the following form:

(16) $$\begin{array}{c} f_{2}\left(\theta ,Q\right)=\det\left(I^{-1}\left(\theta ,Q\right)\right), \end{array}$$

where we now use the acquisition scheme matrix Q instead of the design matrix A as a function argument (see the last paragraph of Section 2.2).

If equal relative variances for a set of K metrics $m_{j}\left(\theta \right)$ are pursued, the objective function should be calculated from the weighted sum of the CRLB diagonal elements:

(17) $$\begin{array}{c} f_{3}\left(\theta ,Q\right)=\sum_{j=1}^{K}w_{j}J{\left(\theta ,Q\right)}_{\left(\mathrm{jj}\right)}, \end{array}$$

with $J{\left(\theta ,Q\right)}_{\left(\mathrm{jj}\right)}$ denoting the *j*th diagonal element of the CRLB and each weight $w_{j}$ equal to the squared reciprocal of the magnitude of the *j*th tissue metric (van den Bos, 2007, pp. 85–86):

(18) $$\begin{array}{c} w_{j}=\frac{1}{m_{j}^{2}\left(\theta \right)}. \end{array}$$

With the weights of Equation (18), we ensure that there is an equal gain in relative precision for each metric $m_{j}\left(\theta \right)$.To ensure acquisition schemes that are optimal for both WM and GM, we minimized the average of the optimality criterion across a representative set of M voxels consisting of both WM and GM (Poot et al., 2010):

(19) $$\begin{array}{c} {\hat{Q}}_{k}=\arg\;\min_{Q}F_{k}\left(Q\right)=\arg\;\min_{Q}\frac{1}{M}\sum_{i=1}^{M}f_{k}\left(\theta_{i},Q\right), \end{array}$$

where ${\hat{Q}}_{k}$ is the optimal acquisition scheme obtained with optimality criterion $f_{k}$ (with $k\in \left\{2,3\right\}$). In addition, we randomly oriented these voxels to avoid tailoring the optimal acquisition scheme to a particular fiber orientation.

### 2.4 Estimators

Here we describe the various estimators that were compared in terms of accuracy and precision. In practice, the DW measurements will be Rician‐distributed, and Basser et al. (1994) showed that the log‐transformed signal intensities can be modeled as:

(20) $$\begin{array}{c} \log\;s=A\theta +\varepsilon , \end{array}$$

where ε is the column vector of independent error terms. The ordinary linear least squares (LLS) estimator of θ is given by:

(21) $$\begin{array}{c} \hat{\theta }={\left(A^{T}A\right)}^{-1}A\;\log\;s. \end{array}$$

It is unbiased under the condition that ε has expectation zero. If the variance of the error terms can be assumed constant across all measurements (an assumption known as homoscedasticity), the LLS estimator is the best linear unbiased estimator of θ (van den Bos, 2007). However, it can be shown that $\mathrm{var}\left(\varepsilon \right)=\sigma^{2}\mathrm{diag}\left({\tilde{s}}^{-2}\right)$, where $\tilde{s}$ is the underlying noise‐free signal vector (Basser et al., 1994). This means that the homoscedasticity assumption does not hold for the log‐transformed data and LLS will have a suboptimal precision. To account for this, a weighted linear least squares (WLLS) estimator was proposed by Basser et al. (1994):

(22) $$\begin{array}{c} \hat{\theta }={\left(AW_{1}A\right)}^{-1}AW_{1}\;\log\;s, \end{array}$$

with $W_{1}$ a diagonal matrix with the reciprocal squares of the elements of the signal vector s on its diagonal:

(23) $$\begin{array}{c} W_{1}=\mathrm{diag}\;\left(s^{-2}\right). \end{array}$$

Alternatively, the iteratively weighted linear least squares (IWLLS) estimator proposed by Salvador et al. (2005) consists of updating the weights of the nth iteration with the signal predictions of the previous iteration, up to some maximum $n_{\max}$:

(24) $$\begin{array}{c} W_{n}=\mathrm{diag}\;{\hat{s}}_{n-1}^{-2}. \end{array}$$

In this work, we set $n_{\max}=2$, as the weight matrix $W_{3}$ did not differ substantially from the previous iteration.

Finally, we define the unweighted nonlinear least squares (NLS) estimator as:

(25) $$\begin{array}{c} \hat{\theta }=\arg\min_{\theta }{\left\|s-\exp\left(A\theta \right)\right\|}_{2}^{2}, \end{array}$$

where ${\left\|.\right\|}_{2}^{2}$ is the squared two‐norm.

Because, in this case, the data are no longer log‐transformed, the variance can be assumed to be constant across all measurements, and weights are not required. In this work, the NLS estimator was initialized with LLS.

### 2.5 Constraints

In this section, we describe the various constraints that can be imposed on the parameters to guarantee their physicality. The mean diffusion tensor $\left\langle D\right\rangle$ is known to be positive semidefinite, or equivalently, for any b‐tensor B, we impose nonnegative diffusivity (Basser & Pajevic, 2007):

(26) $$\begin{array}{c} \left\langle D\right\rangle :B\geq 0. \end{array}$$

Similarly, ℂ is positive definite (Westin et al., 2014):

(27) $$\begin{array}{c} \mathbb{C}:B^{⨂2}\geq 0. \end{array}$$

Note that the constraints described by Equations (26) and (27) have recently been investigated by Herberthson et al. (2021). In this work, we propose two new and more specific nonnegativity constraints on several parameters defined by Westin et al. (2016). The first constraint is a nonnegativity constraint on the isotropic kurtosis:

(28) $$\begin{array}{c} {\mathrm{MK}}_{i}=3\frac{\mathbb{C}:E_{\text{bulk}}}{{\left\langle D\right\rangle }^{⨂2}:E_{\text{bulk}}}\geq 0, \end{array}$$

with $E_{\text{bulk}}$ defined as:

(29) $$\begin{array}{c} E_{b\mathrm{ulk}}=\frac{1}{9}\left(\begin{array}{cccccc} 1 & 0 & 0 & 0 & 0 & 0\\ 0 & 1 & 0 & 0 & 0 & 0\\ 0 & 0 & 1 & 0 & 0 & 0\\ 0 & 0 & 0 & 1 & 0 & 0\\ 0 & 0 & 0 & 0 & 1 & 0\\ 0 & 0 & 0 & 0 & 0 & 1 \end{array}\right). \end{array}$$

A second constraint is nonnegative anisotropic kurtosis:

(30) $$\begin{array}{c} {\mathrm{MK}}_{a}=\frac{6}{5}\frac{\mathbb{C}:E_{\text{shear}}}{{\left\langle D\right\rangle }^{⨂2}:E_{\text{bulk}}}=\mathrm{MK}-{\mathrm{MK}}_{i}\geq 0, \end{array}$$

with MK the mean kurtosis, and with $E_{\text{shear}}$ defined as:

(31) $$\begin{array}{c} E_{\text{shear}}=\frac{1}{9}\left(\begin{array}{cccccc} 2 & -1 & -1 & 0 & 0 & 0\\ -1 & 2 & -1 & 0 & 0 & 0\\ -1 & -1 & 2 & 0 & 0 & 0\\ 0 & 0 & 0 & 3 & 0 & 0\\ 0 & 0 & 0 & 0 & 3 & 0\\ 0 & 0 & 0 & 0 & 0 & 3 \end{array}\right). \end{array}$$

Finally, we also introduce a constraint that enforces monotonic signal decay as was done previously for diffusion kurtosis imaging (Tabesh et al., 2011):

(32) $$\begin{array}{c} \frac{\mathrm{dS}}{\mathrm{db}}\leq 0, \end{array}$$

which implies:

(33) $$\begin{array}{c} \mathbb{C}:g^{⨂4}-b_{\max}\left\langle D\right\rangle :g^{⨂2}\leq 0. \end{array}$$

The three constrained estimators and their associated constraint combinations are described in Table 1. From here on we will prepend the estimator acronym with the letter “C” to indicate the use of constraints and append the number of the constraint combination. For example, the IWLLS estimator combined with constraint combination 3 (Table 1) will be referred to as CIWLLS3, and so on. Note that the second and third constraint combinations (corresponding to CIWLLS2 and CIWLLS3) also implicitly impose semi‐positive definiteness on ℂ (i.e., the constraint described by Equation 27). Moreover, the constraint combination corresponding with CIWLLS2 is similar in spirit to the constraints imposed by Tabesh et al. (2011) for DKI.

**TABLE 1:** The various constrained estimators used to estimate the 28 tensor parameters of the signal model

Estimator	$\left\langle D\right\rangle :B\geq 0$	$\mathbb{C}:B^{⨂2}\geq 0$	${\mathrm{MK}}_{i}\geq 0$	${MK}_{a}\geq 0$	$\frac{\mathrm{dS}}{\mathrm{db}}\leq 0$	Reference
CIWLLS1	Yes	Yes	No	No	No	Herberthson et al. (2021)
CIWLLS2	Yes	Yes*	No	No	Yes	Similar to Tabesh et al. (2011)
CIWLLS3	Yes	Yes*	Yes	Yes	Yes	–

*Note*: The asterisk indicates that the constraint is imposed implicitly as a consequence of the other constraints.

## 3 MATERIALS AND METHODS

### 3.1 Optimal experimental design

Following the theory in Section 2.3, we generated two optimized acquisition schemes each containing 120 DW samples: ${\hat{Q}}_{2}$ which minimizes the determinant of the covariance matrix of the raw DTD parameters (see Equation 16), and ${\hat{Q}}_{3}$ which minimizes the weighted average of the variances of derived scalar measures (see Equation 17) and compare them to a naïve acquisition scheme of equal length $Q_{1}$ (see Table 2 for the specifications of this acquisition scheme). The set of metrics for which the acquisition scheme ${\hat{Q}}_{3}$ was optimized using Equation (17) was MD, μFA, MK_i_, and MK_a_. The naïve reference scheme $Q_{1}$ was based on the scheme in Szczepankiewicz, Hoge, and Westin (2019) from which we took the distribution of *b* values and b‐tensor schemes, but reduced the total number of samples to 120 to ensure fair comparison. For each set of DW samples corresponding to a specific *b*‐value and b‐tensor shape combinations, we additionally applied electrostatic repulsion to the principal b‐tensor axes to ensure rotational invariance of $Q_{1}$.As the CRLB depends on the underlying tissue parameters θ, a random selection of 2000 representative voxels (consisting of WM and GM) were selected from the open data set provided by Szczepankiewicz, Hoge, and Westin (2019). To ensure rotational invariance of the optimized scheme, the gradient scheme of each voxel was randomly reoriented. For each voxel, we then estimated θ from the densely sampled open dataset provided by Szczepankiewicz, Hoge, and Westin (2019) (N=377 DW samples, see Table S1 for acquisition details) using the CIWLL3 estimator.

**TABLE 2:** Optimal experimental design: Distribution of the 120 DW samples across the *b* values and b‐tensor shapes of the naive acquisition scheme $Q_{1}$, the acquisition scheme ${\hat{Q}}_{2}$ obtained from optimizing the determinant of the CRLB matrix and the acquisition scheme ${\hat{Q}}_{3}$ obtained from optimizing the weighted trace of the CRLB of MD, MK_i_, MK_a_, and μFA (Section 3.1)

Acquisition scheme	$Q_{1}$			${\hat{Q}}_{2}$			${\hat{Q}}_{3}$		
b (ms/μm^2^ )	PTE	STE	LTE	PTE	STE	LTE	PTE	STE	LTE
0.1	3	17	3	–	–	6	7	–	9
0.7	3	17	3	–	–	–	–	–	–
0.8	–	–	–	–	–	30	9	–	50
1.4	5	17	5	–	–	–	–	–	–
2	15	17	15	36	–	48	–	30	15

The starting point for minimizing Equation (19) was obtained by generating 120 directions distributed uniformly on the unit sphere with electrostatic repulsion and initializing the *b* values b and b‐tensor anisotropies $b_{\Delta }$ by drawing them randomly from a uniform distribution such that $0.1<b<2\;s/\mu m^{2}$ and $-0.5\leq b_{\Delta }\leq 1$. We minimized Equation (20) with respect to b and $b_{\Delta }$ for the two objective functions $f_{2}$ and $f_{3}$ (described by Equations (16) and (17)) using MATLAB's built‐in *patternsearch* function, while keeping the directions G (see Equation 12) fixed. Fixing the directions of the b‐tensors has several advantages. First, it greatly reduces the number of parameters that have to be optimized, reducing the computational cost and making the optimization less prone to local minima. Second, it ensures that the resulting acquisition scheme will be more or less rotationally invariant. Nevertheless, the resulting optimization is not convex. For this reason, we used the Direct Search algorithm from MATLAB's Global Optimization Toolbox (*patternsearch*).

To ensure a shell‐wise acquisition, we grouped all samples of the optimized acquisition scheme with similar *b* values and b‐tensor shapes together. To ensure rotational invariance, the b‐tensor principal axes of each group of DW samples were then redistributed uniformly on the unit sphere using electrostatic repulsion.

### 3.2 Acquisition and preprocessing

An in vivo data set of a healthy human brain was acquired from a 29‐year‐old male volunteer after obtaining written informed consent. It contains five repetitions of each acquisition scheme ($Q_{1},{\hat{Q}}_{2}$, and ${\hat{Q}}_{3})$, resulting in a total of 3 × 5 × 120 = 1800 DW samples. We used a Siemens MAGNETOM 3T Prisma system with a custom pulse sequence based on a diffusion‐weighted spin‐echo that supports free waveform encoding (FWF, version 1.19 s), enabling PTE, STE, and LTE (Szczepankiewicz, Sjölund, et al., 2019). The imaging parameters used were: TR = 4 s, TE = 91 ms, FOV = 220 × 220 × 62.5 mm, matrix = 88 × 88 × 25, isotropic voxel size = 2.5 mm^3^, partial‐Fourier = 7/8, bandwidth = 1960 Hz/px, echo spacing = 0.6 ms. Furthermore, we used in‐plane acceleration iPAT = 2 with GRAPPA reconstruction without simultaneous multiband acquisition (SMS). We preprocessed it with a state‐of‐the‐art pipeline consisting of denoising (Veraart et al., 2016), Gibbs‐ringing correction (Kellner et al., 2016), and extrapolation‐based affine motion and distortion correction (Nilsson et al., 2015). Denoising and Gibbs‐ringing correction were performed using MRtrix3 (Tournier et al., 2019).

### 3.3 Simulations

To evaluate the different estimators, we compared the bias, the standard deviation, and the root‐mean‐squared error (RMSE) of the unconstrained LLS, WLLS, IWLLS, and NLS estimators with whole‐brain Monte‐Carlo simulations. We estimated the ground truth θ from 2000 voxels (consisting of both WM and GM) across the entire brain using the CIWLLS3 estimator, using the open data set provided by Szczepankiewicz, Hoge, and Westin (2019). The noise‐free signal was generated with the forward model (Equation 8) using acquisition scheme ${\hat{Q}}_{3}$. To add Rician noise, a realistic σ was estimated from the open dataset by averaging a WM‐masked noise map, obtained from the denoising approach proposed by Veraart et al. (2016), resulting in an average SNR of 25 at *b* = 0 ms/μm^2^ in WM.

To evaluate the effect of the different constraints, we additionally compared the bias, the standard deviation, and the RMSE of the estimators IWLLS and CIWLLS1 to CIWLLS3 at an SNR of 25 in the same WM and GM voxels.

### 3.4 Real data experiments

To validate the precision improvement of the different acquisition schemes or real data, we used the five repetitions of each acquisition scheme and empirically calculated the standard deviation of several scalar DTD parameters. To avoid the unwanted effects of residual misregistration and CSF pulsation between those five repetitions, we restricted this analysis to a conservative WM mask, steering away from the ventricles and outside of the brain where misregistration could adversely affect the estimation of precision.

To demonstrate the performance of the LLS, WLLS, NLS, and IWLLS estimators on real data, we compared the estimates obtained from a single, undenoised repetition of acquisition scheme ${\hat{Q}}_{3}$ to high‐precision “benchmark values.” These benchmark values were obtained as follows. First, all 1800 DW samples available across repetitions and acquisition schemes were concatenated and denoised simultaneously. Using the full dataset as a reference maximizes data redundancy which will boost the efficacy of random matrix denoising (Veraart et al., 2016). We then estimated the benchmark parameters from this large and highly denoised data set using the IWLLS estimator.^1^ The deviation in the estimation of MD, FA, μFA, MK_i_, MK_a_, and C_c_ was then evaluated by applying the LLS, WLLS, NLS, and IWLLS estimators to the same single repetition without denoising.

To demonstrate the performance of the IWLLS and CIWLLS1 to CIWLLS3 estimators on noisy real data, the standard deviation in the estimation of MD, FA, μFA, MK_i_, MK_a_, and OP was empirically calculated across to the five repetitions acquired with acquisition scheme ${\hat{Q}}_{3}$ without denoising.

## 4 RESULTS

### 4.1 Optimal experimental design

Table 2 depicts the distribution of DW samples across the *b* values and b‐tensor shapes of the naive acquisition scheme $Q_{1}$, and that of the optimized acquisition schemes ${\hat{Q}}_{2}$ and ${\hat{Q}}_{3}$ after clustering (Figure S1 shows the optimized schemes *before* clustering). Interestingly, despite the optimization being allowed to select *any* axially symmetric b‐tensor, the optimal b‐tensors were strongly clustered around the quintessential shapes LTE, PTE, and STE. Given the strong clustering, we opted to limit the final acquisition scheme to only use pure LTE, PTE, and/or STE at discrete *b* values as this facilitates adoption.

Compared to the naive scheme $Q_{1}$, fewer (b, $b_{\Delta })$ clusters can be observed in both ${\hat{Q}}_{2}$ and ${\hat{Q}}_{3}$. Acquisition scheme ${\hat{Q}}_{2}$ has 6, 30, and 48 LTE samples at *b* values 0.1, 0.8, and 2 ms/μm^2^, respectively, and 36 PTE samples at a *b*‐value of 2 ms/μm^2^. Acquisition scheme ${\hat{Q}}_{3}$ has 9, 50, and 15 LTE samples at *b* values 0.1, 0.8, and 2 ms/μm^2^, respectively, 30 STE samples at a *b*‐value of 2 ms/μm^2^, and 7 and 9 PTE samples at *b* values 0.1 and 0.8 ms/μm^2^.

**FIGURE 1:**
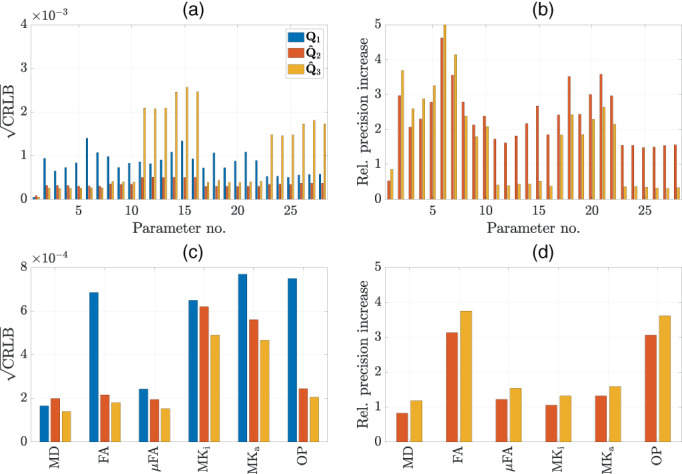
Optimal experimental design: (a) the median CRLBs across 2000 WM and GM voxels of the raw DTD tensor parameters θ at SNR = 15 for the naive acquisition $Q_{1}$, ${\hat{Q}}_{2}$ and ${\hat{Q}}_{3}$. (b) The median relative precision increase of the raw DTD tensor parameters of the optimized acquisition schemes compared to the naive scheme $Q_{1}$. (c) The CRLBs of the tissue metrics at SNR = 15. The units of MD are in μm/ms^2^. (d) The relative precision increase of the tissue metrics of the optimized schemes compared to the naive scheme

Figure 1 shows the lower bounds on the precision of the raw DTD parameters as well as for the derived scalar metrics for the optimized schemes as well as for the naive acquisition scheme $Q_{1}$. Figure 1a depicts the CRLB at an SNR of 15 of the 28 parameters contained in θ. For the naive scheme $Q_{1}$, the CRLBs of the tensor parameters (i.e., parameters 2–28) are comparable in magnitude. For acquisition scheme ${\hat{Q}}_{2}$, the CRLBs of all parameters are consistently lower than those of the naive scheme $Q_{1}$. For acquisition scheme ${\hat{Q}}_{3}$, parameters 1–10 and 17–22 have a lower CRLB compared to the naive scheme, whereas parameters 11–16 and 23–28 have a substantially higher CRLB than those of the naive scheme.

Figure 1b shows the relative precision gain for each parameter with respect to the naive scheme:

(34) $$\begin{array}{c} p_{\text{gain}}\left(Q_{i}\right)=\frac{p_{\mathrm{abs}}\left(Q_{i}\right)}{p_{\mathrm{abs}}\left(Q_{1}\right)}\;\text{with}\;p_{\mathrm{abs}}\left(Q_{i}\right)=\frac{1}{\sqrt{\text{CRLB}\left(Q_{i}\right)}} \end{array}$$

with values lower than 1 indicating a drop and values higher than 1 indicating a gain in precision compared to the reference scheme. For acquisition scheme ${\hat{Q}}_{2}$, a precision loss of a factor of 0.5 can be observed for parameter 1 (i.e., S_0_), whereas precision gains of a factor of 1.5 up to 3.5 can be observed for the remaining parameters. Acquisition scheme ${\hat{Q}}_{3}$ shows a precision loss of a factor of 0.85 for parameter 1, precision gains of a factor of 1.7 up to 4 for parameters 2–10 and 17–22, and a precision loss of a factor of 0.3 for parameters 11–16 and 23–28. The mean precision gains over all parameters are 2.32 and 1.67 for acquisition schemes ${\hat{Q}}_{2}$ and ${\hat{Q}}_{3}$, respectively.Figure 1c shows the CRLBs of the scalar DTD parameters mean diffusivity (MD), fractional anisotropy (FA), microscopic FA (μFA), isotropic kurtosis (MK_i_), the anisotropic kurtosis (MK_a_), and the order parameter (OP). The definition of these parameters can be retrieved from Westin et al. (2016). Although the CRLBs of the naive scheme do not vary considerably in magnitude across the raw DTD parameters (compared to the other two acquisition schemes), the CRLBs of the metrics can vary considerably in magnitude. Compared to the naive acquisition scheme $Q_{1}$, acquisition scheme ${\hat{Q}}_{2}$ has a higher CRLB for MD but lower CRLBs for the remaining metrics, whereas acquisition scheme ${\hat{Q}}_{3}$ has lower CRLBs for all metrics.

Figure 1d shows the relative precision gain with respect to the naive scheme for the metrics MD, FA, μFA, MK_i_, MK_a_, and OP. Compared to the naive acquisition scheme, acquisition scheme ${\hat{Q}}_{2}$ exhibits a precision loss of a factor 0.8 for MD. For the remaining metrics, a considerable precision gain ranging from a factor of 1.1 for MK_i_ up to factor of 3.1 for FA is observed. Acquisition scheme ${\hat{Q}}_{3}$ exhibits a larger precision gain than acquisition scheme ${\hat{Q}}_{2}$, ranging from a factor of 1.2 for MD up to a factor of 3.8 for FA. The mean precision gains over all metrics are 1.77 and 2.17 for acquisition schemes ${\hat{Q}}_{2}$ and ${\hat{Q}}_{3}$, respectively.

### 4.2 Simulations

**FIGURE 2:**
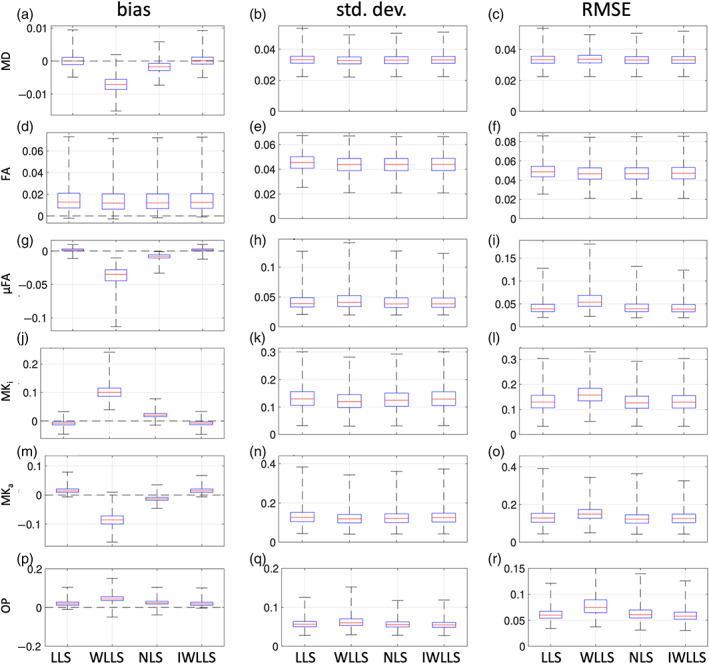
Simulations: Bias, standard deviation, and RMSE of the different estimators for: MD (in μm/ms^2^) (a–c), FA (d–f), μFA (g–i), MK_i_ (j–l), MK_a_ (m–o), and OP (p–r) of WM and GM voxels across the whole brain at SNR = 25. The distance between the whiskers is five times the interquartile width

Figure 2 shows boxplots of the bias, standard deviation, and RMSE in the estimation of MD, FA, μFA, MK_i_, MK_a_, and OP using the LLS, WLLS, NLS, and IWLLS estimators, based on 400 noise realizations (SNR = 25) in whole‐brain WM and GM voxels. The forward signal was generated with acquisition scheme ${\hat{Q}}_{3}$.The bias of IWLLS and LLS is lower compared to WLLS and NLS for MD, μFA, and OP (Figure 2a,g,m,p). For FA, the bias is comparable for all estimators (Figure 2d), whereas for MK_a_ and MK_i_ the bias of IWLLS and LLS are comparable and lower than that of NLS and WLLS, respectively (Figure 2j,m).

The standard deviation in the estimation of MD is lowest for WLLS and slightly higher but comparable for LLS, NLS, and IWLLS (Figure 2b). For MK_i_ and MK_a_, the standard deviation is lower for WLLS and NLS (Figure 2k,n), whereas, for FA, LLS, NLS, and IWLLS have a lower standard deviation. For μFA and OP, LLS, NLS, and IWLLS have a standard deviation lower than WLLS (Figure 2h,q).

The RMSE in the estimation of MD is lower for IWLLS and NLS (Figure 2c). For FA, the RMSE is comparable and lower for WLLS, NLS, and IWLLS (Figure 2f) than that of LLS. For μFA, MK_i_, MK_a_, and OP the RMSE is lowest and comparable for LLS, NLS, and IWLLS and the RMSE for WLLS is highest (Figure 2i,l,o,r). The results of the same experiment but at an SNR of 15 can be appreciated in Figure S2.

In summary, although WLLS offers a slightly more precise estimation of some parameters, it also has a considerable bias for almost all parameters considered (with the median relative bias ranging from −0.83% to 32.74% for MD and MK_i_, respectively). Conversely, LLS and IWLLS exhibit a much smaller bias (with the medial relative bias ranging from 0.01% up to 7.42% for MD and MK_i_, respectively), while IWLLS simultaneously offers a slightly improved precision for some parameters compared to LLS. In terms of RMSE, the LLS and IWLLS estimators are comparable, whereas WLLS consistently performs worse.

**FIGURE 3:**
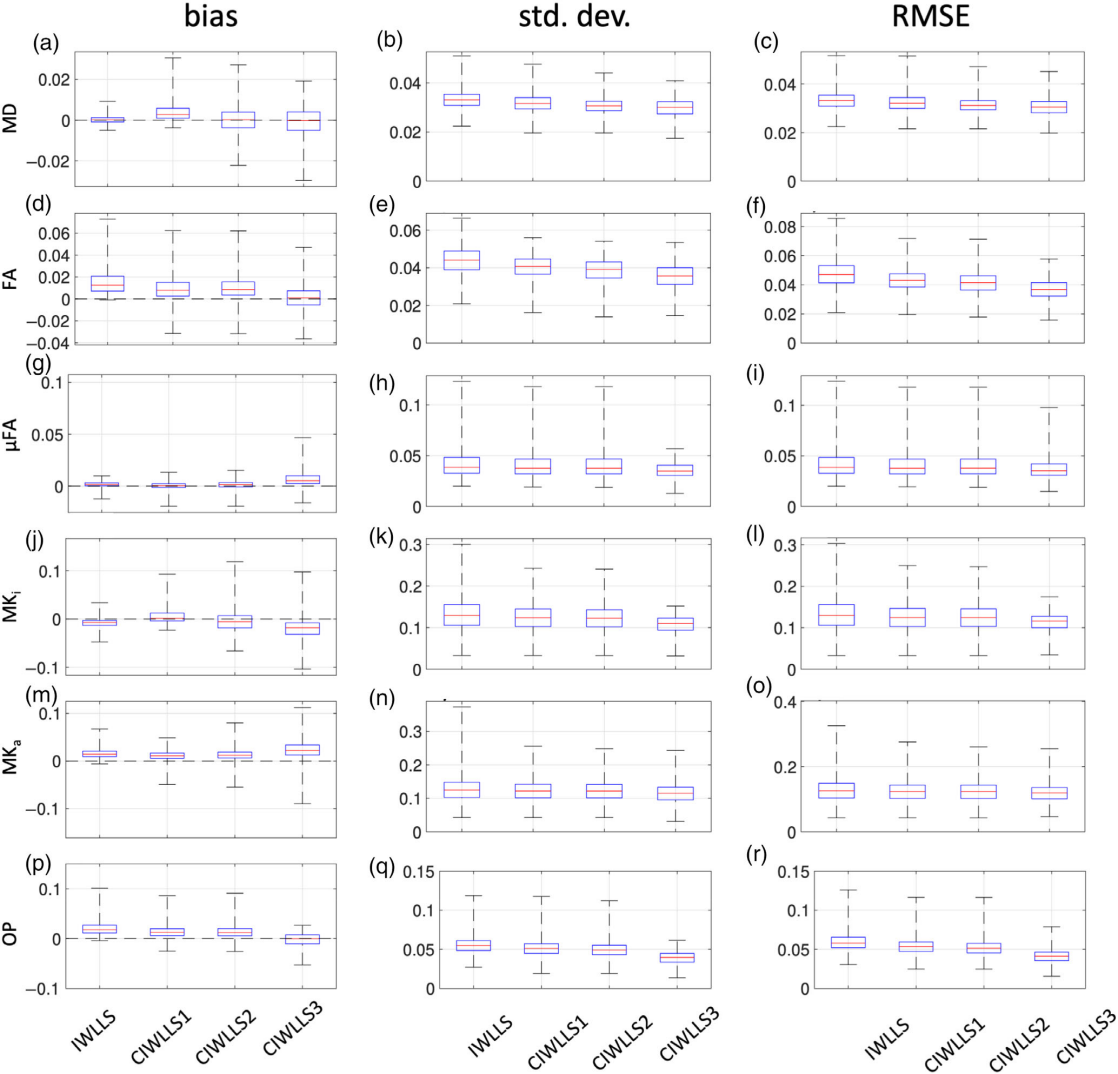
Simulations: Bias, standard deviation, and RMSE of the IWLLS and CIWLLS1 to CIWLLS3 estimators for MD (in μm/ms^2^) (a–c), FA (d–f), μFA (g–i), MK_i_ (j–l), MK_a_ (m–o), and OP (p–r) of WM GM voxels across the whole at SNR = 25. The distance between the whiskers is five times the interquartile width

Figure 3 shows boxplots of the bias, standard deviation, and RMSE in the estimation of MD, FA, μFA, MK_i_, MK_a_, and OP using the IWLLS and CIWLLS1 to CIWLLS3 estimators, based on 400 noise realizations (SNR = 25) in whole‐brain WM and GM voxels.

For MD, IWLLS and CIWLLS2 and CIWLLS3 are least biased, followed by CIWLLS1 (Figure 3a). For FA and OP, CIWLLS3 exhibits the lowest bias, followed by CIWLLS1, CIWLLS2, and IWLLS (Figure 3d,p). For μFA, IWLLS, CIWLLS1, and CIWLLS2 are least biased, whereas CIWLLS3 exhibits a slight overestimation (Figure 3g). For MK_i_, CIWLLS1 is the least biased, followed by IWLLS and CIWLLS2, and lastly CIWLLS3 (Figure 3j). For MK_a_, IWLLS, CIWLLS1, and CIWLLS2 are least biased, whereas CIWLLS3 is more biased (Figure 3m).

The standard deviation in the estimation of MD is highest for IWLLS, followed by CIWLLS1 and CIWLLS2, with CIWLLS3 having the lowest standard deviation (Figure 3b). For FA and OP, IWLLS has the highest standard deviation, whereas the standard deviation becomes progressively lower going from CIWLLS1 to CIWLLS3 (Figure 3e,q). For μFA, MK_i_, MK_a_, IWLLS, CIWLLS1, and CIWLLS2 have a higher and comparable standard deviation, whereas CIWLLS3 has the lowest standard deviation (Figure 3h,k,n). In terms of RMSE, similar trends can be observed as those seen for the standard deviation.

In summary, in terms of bias, there is no clear best estimator, as this varies across the different metrics considered, but its magnitude is small compared to that of the unconstrained estimators. For the standard deviation, the CIWLLS3 estimator shows the best performance, and IWLLS the worst. In terms of RMSE, the CIWLLS3 estimator performs best.

### 4.3 Real data experiments

**FIGURE 4:**
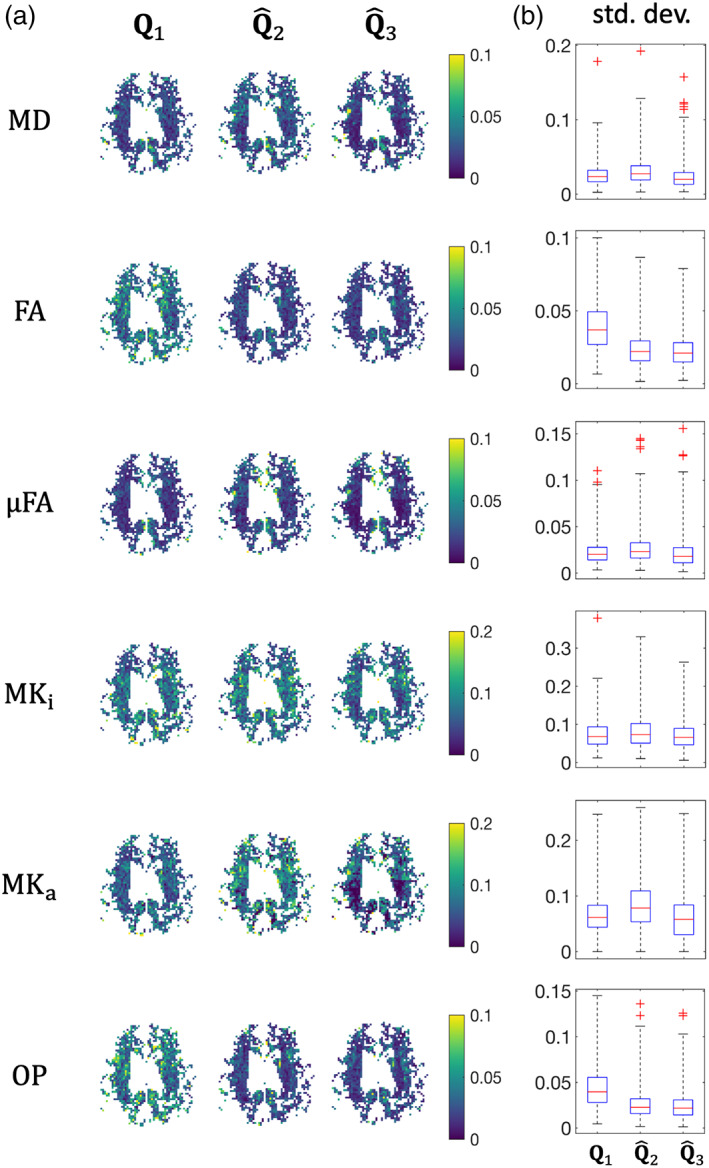
Real data: (a) empirically calculated standard deviations of various metrics based acquisitions $Q_{1}$, ${\hat{Q}}_{2}$, and ${\hat{Q}}_{3}$ in an axial slice. (b) Boxplots of the distribution of standard deviations in the axial slices depicted in (a). The distance between the whiskers is five times the interquartile width. The units of MD are μm/ms^*2*^

**FIGURE 5:**
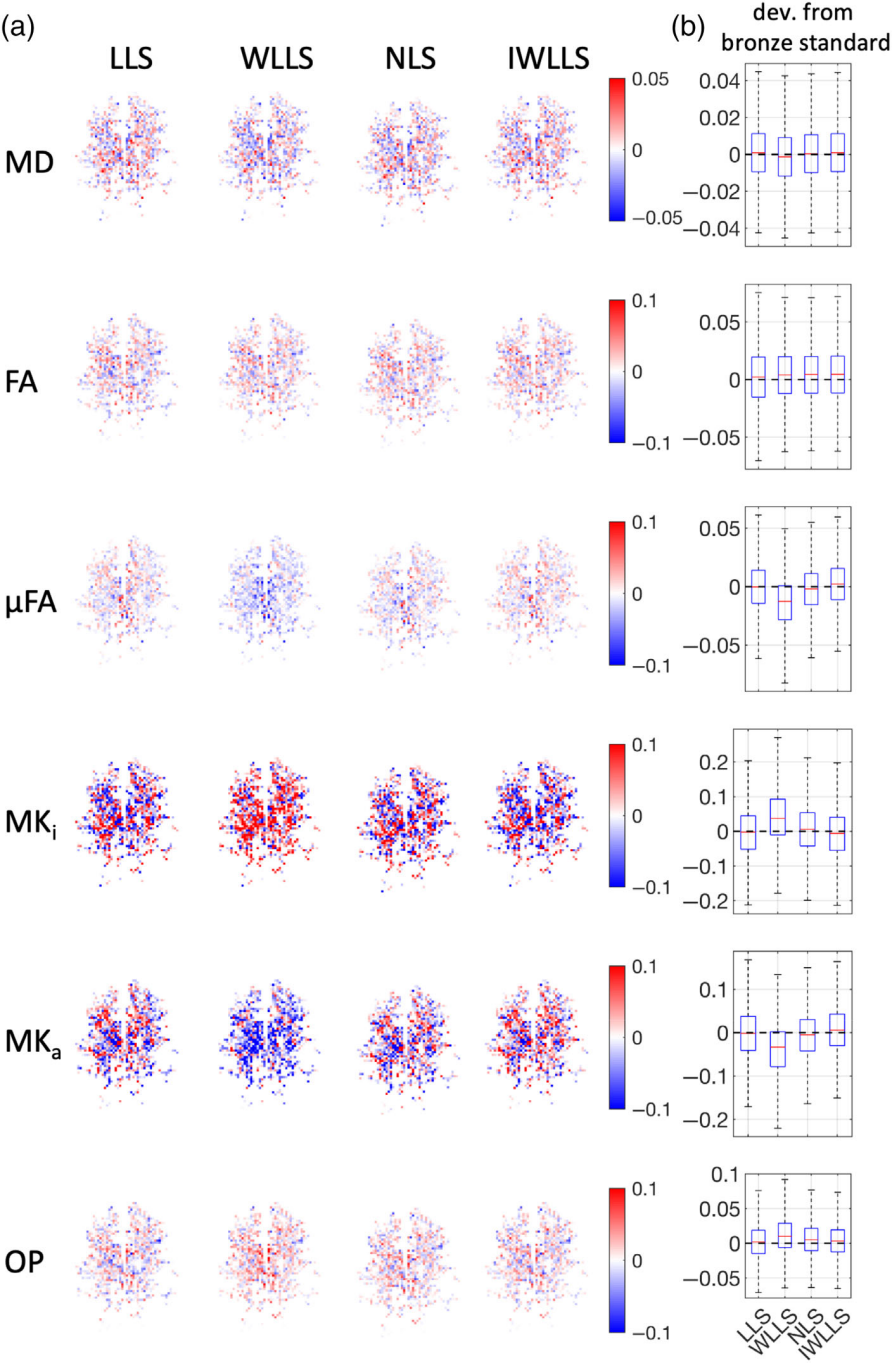
Real data: (a) deviation from the benchmark values for the different estimators (based on acquisition scheme ${\hat{Q}}_{3}$). (b) Boxplots of the slices depicted in (a). The distance between the whiskers is five times the interquartile width. The units of MD are μm/ms^2^

Figure 4 shows axial maps and boxplots of the empirically calculated standard deviations of several metrics for the different acquisition schemes. Similar trends as in Figure 1 can be observed. For MD, acquisition scheme ${\hat{Q}}_{2}$ has a lower precision than acquisition schemes $Q_{1}$ and ${\hat{Q}}_{3}$, corresponding with the predicted SNR loss. For FA and OP, a considerable increase in precision can be observed for acquisition schemes ${\hat{Q}}_{2}$ and ${\hat{Q}}_{3}$, compared to the naive. For MK_i_ and MK_a_, acquisition scheme ${\hat{Q}}_{2}$ exhibits a lower precision compared to the naive scheme $Q_{1}$ and ${\hat{Q}}_{3}$. For μFA, acquisition scheme ${\hat{Q}}_{3}$ has the highest precision, followed closely by acquisitions ${\hat{Q}}_{2}$ and $Q_{1}$, respectively. Overall, acquisition scheme ${\hat{Q}}_{3}$ has the highest precision for all parameters considered. The median precision gains overall metrics are 0.90 and 1.16 for acquisition schemes ${\hat{Q}}_{2}$ and ${\hat{Q}}_{3}$, respectively.Figure 5 depicts an axial slice of the deviation from the benchmark values in WM and GM voxels of the LLS, WLLS, NLS, and IWLLS estimators when applied to noisy real data. Similar trends can be observed as predicted in the simulations. The WLLS estimator tends to underestimate MD, μFA, and MK_a_, whereas it tends to overestimate MK_i_ and OP.

**FIGURE 6:**
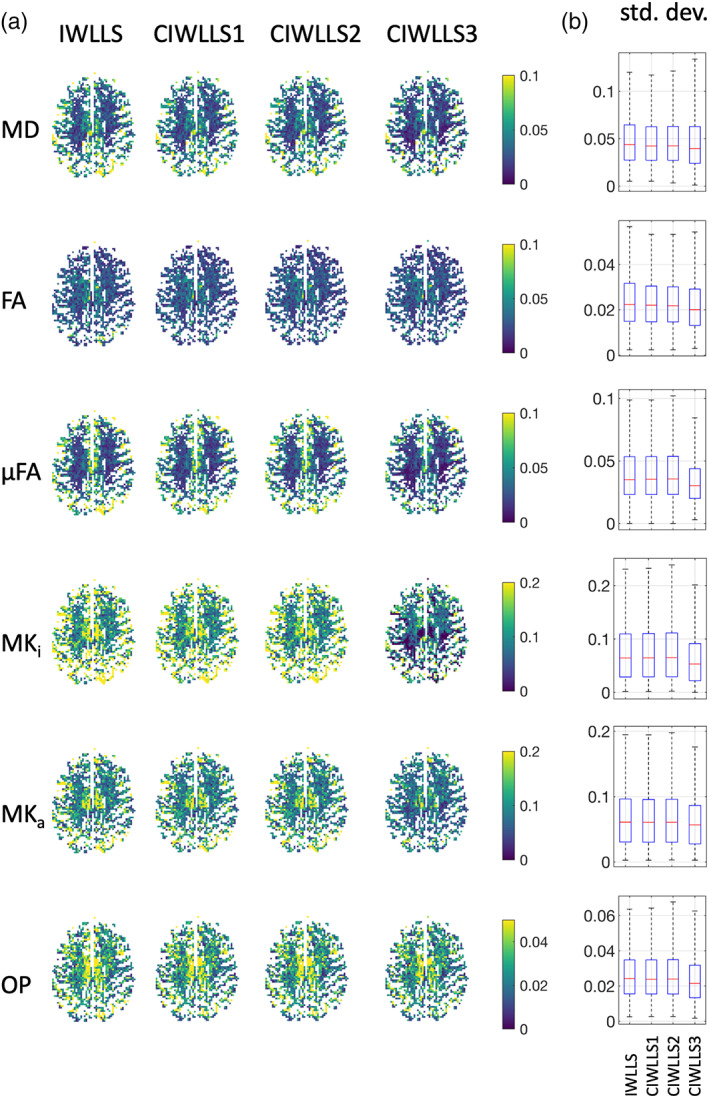
Real data: (a) standard deviation of the IWLLS and CIWLLS1 to CIWLLS3 estimators (based on five repetitions of acquisition scheme ${\hat{Q}}_{3}$). (b) Boxplots of the slices depicted in (b). The distance between the whiskers is five times the interquartile width. The units of MD are μm/ms^2^

Figure 6 depicts the standard deviation of the IWLLS and CIWLLS1 to CIWLLS3 estimators. Across the metrics MD, FA, μFA, MK_i_, and MK_a_, a clear increase in precision can be observed as the constraints become more stringent. For OP, the increase in precision is only minor.

**FIGURE 7:**
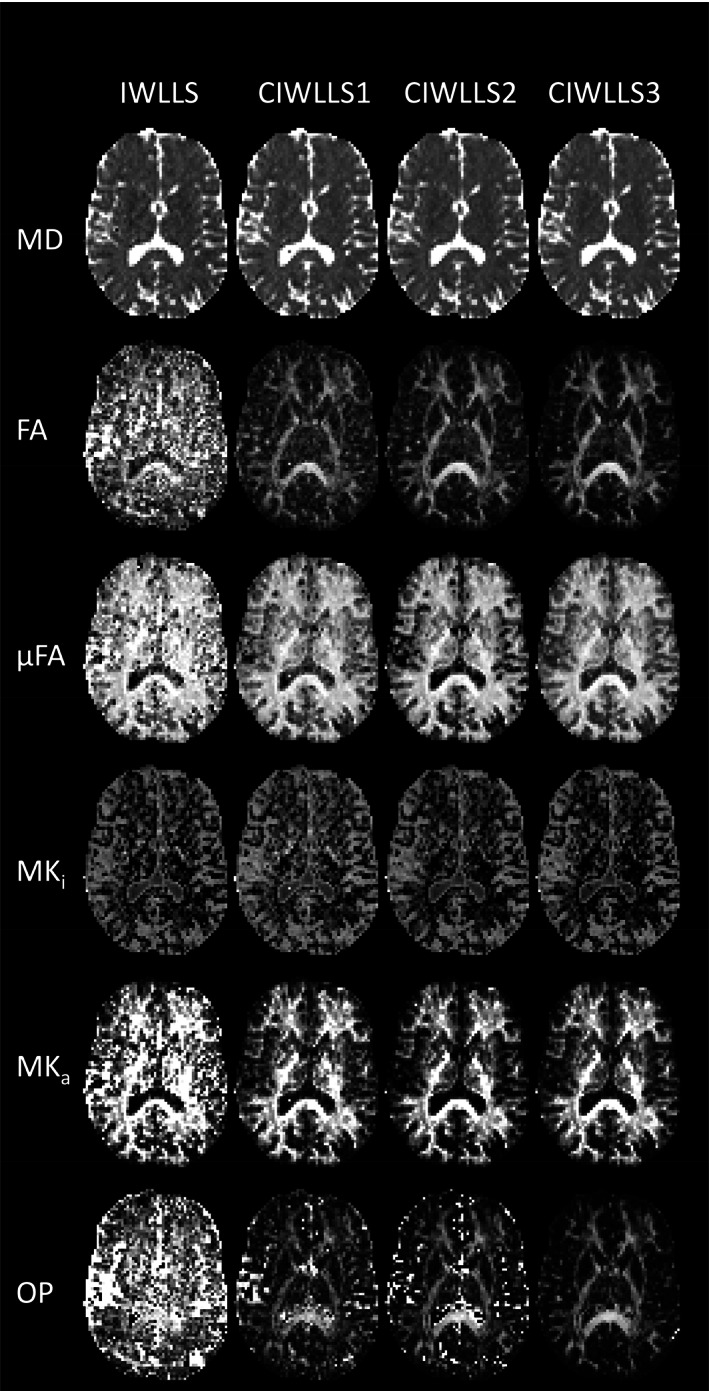
Real data: Axial map of scalar DTD metrics estimated just 60 q‐space samples using the IWLLS and CIWLLS1 to CIWLLS3 estimators

Figure 7 shows an axial map of metrics obtained with the IWLLS and CIWLLS1 to CIWLLS3 estimators, estimated from a single repetition acquired with only half the data (i.e., 60 DW samples). Compared to IWLLS, the constrained estimators progressively (i.e., going from CIWLLS1 to CIWLLS3) reduce the number of voxels with spurious fitting results, especially for FA, μFA, MK_a_, and OP, and to a lesser extent also in MD and MK_a_.

## 5 DISCUSSION

In this work, we have aimed to improve the precision and accuracy of metrics estimated from QTI data. We have found that a precision‐maximizing sampling scheme, combined with an iteratively reweighted linear least squares estimator provides the best results in terms of accuracy and precision.

### 5.1 Implications for the acquisition of QTI data

The sampling strategy has a considerable impact on the maximally attainable precision of the raw DTD tensor parameters, which directly affects the precision of the scalar DTD parameters (i.e., MD, FA, μFA, MK_i_, MK_a_, and OP). Optimal experiment design for QTI has been explored previously in the work by Coelho et al. (2019), where the same criterion as the one described by Equation (16) was used (Section 2.3), that is, the determinant of the CRLB matrix of the raw DTD tensor parameters. Although we used a different data set for the prior distribution of WM and GM voxels, our results align remarkably well with their findings. In their work, an acquisition scheme optimized for the maximally precise estimation of the raw DTD parameters requires 75% of LTE and 25% of PTE samples at *b* values of 0.1, 0.7, and 2 ms/μm^2^. Similarly, in this work, we found 70% of LTE and 30% of PTE samples at *b* values 0.1, 0.8, and 2 ms/μm^2^ (see ${\hat{Q}}_{2}$ in Table 2). However, their analysis only considered the impact on the raw DTD tensor parameters, not the precision of the scalar DTD parameters.

In this work, we found that, compared to a naive sampling scheme, a sampling scheme that maximizes the precision of the raw DTD tensor parameters can even hurt the precision of the scalar DTD parameters. Indeed, as is shown in Figure 1d, there is a relative precision loss down to a factor of 0.83 for MD. The remaining parameters have a relative precision gain ranging from a factor of 1.05 for MK_i_, up to a factor of 3.13 for FA. To avoid the loss in precision for MD, we minimized a criterion based on the weighted trace of the CRLB matrix of the scalar DTD parameters MD, μFA, MK_i_, and MK_a_, described by Equation (17) (Section 2.3). Using this criterion, the sampling scheme ${\hat{Q}}_{3}$ was obtained, which consisted of 13% PTE samples, 25% STE, and 62% LTE samples. The *b* values were again 0.1, 0.8, and 2 ms/μm^2^. This sampling strategy resulted in relative precision increases ranging from a factor of 1.18 for MD, up to a factor of 3.75 for FA, and outperforming acquisition ${\hat{Q}}_{2}$ across the board. We validated these three acquisition schemes with real data and, however, we observed similar trends in precision (Figure 4).

Note that ${\hat{Q}}_{2}$ allocated most samples to the highest *b*‐value shells, as is customary in multi‐shell acquisition schemes, because these contain higher angular frequencies. ${\hat{Q}}_{3},$ on the other hand, allocated more samples to the intermediate shells, which might be counter intuitive. It is important to realize that ${\hat{Q}}_{2}$ is a set of DW samples that minimizes the determinant of the covariance matrix of *the raw QTI parameters* (1 + 6 + 21 = 28 parameters). These parameters encode not only the radial dependency, but also the angular dependency of the signal. To precisely capture this angular dependency, it makes sense that ${\hat{Q}}_{2}$ favors a high number of directions at the outer shell. Moreover, because STE contains no angular information, ${\hat{Q}}_{2}$ does not contain any STE measurement and PTE measurements are favored instead. By contrast, ${\hat{Q}}_{3}$ minimizes the uncertainty of the (rotationally invariant) scalar parameters. As ${\hat{Q}}_{3}$ does not rely on (complete) angular information, it makes sense that fewer directions are required at the outer shell. Moreover, angular contrast (from PTE and LTE) in ${\hat{Q}}_{2}$ is traded in for shape contrast (STE) in ${\hat{Q}}_{3}$.The optimal acquisition scheme ${\hat{Q}}_{3}$ is closely related to recent work by Arezza et al. (2021), which optimized a sequence specifically for μFA estimation based on powder‐averaged STE and LTE images. They looked for the *b*‐value, and proportion of STE to LTE samples at this *b*‐value, that results in maximal contrast‐to‐noise‐ratio between the STE signal and the (powder‐averaged) LTE signal, as, under certain assumptions (variance in ADC is negligible compared to the variance in μFA and ADC does not depend on tensor shape), this provides a proxy for the precision of μFA. Note that, because of the way their optimization criterion is constructed, it is limited to STE and LTE samples only, whereas our approach explores all axially symmetric b‐tensor shapes. Moreover, their approach can only make recommendations about the proportion of STE to LTE samples *at a single b‐*value and does not optimize a comprehensive multi‐shell acquisition scheme like in our work. Despite these differences, the recommendations of Arezza et al. (2021) are in good agreement with ours. First, they found an optimal *b*‐value of 2 ms/μm^2^, which corresponds to the outer shell found in our optimized scheme. Second, they found the optimal proportion of STE to LTE samples at this *b*‐value to be approximately 1.7, which is close to the proportion of 2 found in ${\hat{Q}}_{3}$ for the *b* = 2 ms/μm^2^ shell. About the number of *b* = 0 ms/μm^2^ samples and intermediate *b* values, the optimization criterion in Arezza et al. (2021) provides no recommendations due to its single‐shell nature.

Our results show that the choice of optimality criterion has a nontrivial effect on the maximally attainable precision. When using the criterion based on the determinant of the CRLB, all DTD tensor parameters simultaneously have a higher precision compared to the naive sampling scheme, and the precision for the scalar DTD parameters FA, μFA, MK_i_, MK_a_, and OP also increases. However, the precision of MD is lower than that of the naive acquisition scheme. By contrast, a criterion that is tailored to maximize the precision for the scalar DTD parameters of interest results in the proposed optimal acquisition scheme ${\hat{Q}}_{3}$, which provides a precision increase for all scalar DTD parameters compared to the naive acquisition scheme. For studies that employ these parameters as a biomarker, we recommend using acquisition scheme ${\hat{Q}}_{3}$.The proposed optimal acquisition scheme consists of only 120 DW samples, which translates to a scanning time of only 8 min. Note that we did not employ SMS, which could reduce the scanning time down to almost 4 min.

### 5.2 Impact of the estimator on the accuracy and precision of tissue metrics

The choice of the estimator plays an important role in the accuracy and precision of the scalar DTD parameters. Previously, Westin et al. (2016) estimated the DTD tensor parameters using the WLLS estimator with the squared reciprocals of the noisy DW signal as weights to account for the heteroscedasticity of the data. In this work, we have observed a bias when using these weights to estimate the scalar DTD parameters (Figure 2). To combat this, the IWLLS estimator, which iteratively updates the weights of the WLLS estimator using the inverse squares of the predicted signal, considerably reduces the bias while still offering some of the benefits of improved precision when using weighted linear estimation. Indeed, compared to LLS, WLLS and IWLLS offer a median relative precision increase up to a factor of 1.03 for FA. Furthermore, when using WLLS, we observed median relative biases up to a factor of 1.328 and 1.195 in the estimation of MK_i_ and OP, respectively, whereas the IWLLS estimator exhibited a median relative bias in the estimation of these parameters down and up to factors of −0.024 and 0.074, respectively. This is in accordance with previous observations for DTI and DKI, where the WLLS estimator is known to have improved precision over the conventional LLS estimator when using these weights, but at the cost of a severe bias (Veraart et al., 2013). In addition, in terms of bias, we found that LLS and IWLLS outperform the NLS estimator, and in terms of precision the LLS, IWLLS, and NLS estimators are comparable (Figure 2).

### 5.3 Impact of constraints on the accuracy and precision of tissue metrics

The choice of constraints affects the accuracy and precision of the estimated parameters. Indeed, guaranteeing the physicality of the estimates of the tensor parameters reduces the size of the search space, thus improving the noise resilience of the estimator. Imposing semi‐positive definiteness on the diffusion and covariance tensors has recently been proposed by Herberthson et al. (2021), and they demonstrated that these constraints can substantially improve the quality of scalar DTD parameter maps, even on very limited data.

Our approach goes one step further, as we impose additional constraints on the scalar DTD metrics MK_i_ and MK_a_, as well as requiring the signal to decrease monotonically as a function of the *b*‐value. Compared to the unconstrained estimator, the constraints that had the largest impact on the precision were the semi‐positive definiteness of the diffusion and covariance tensors, as well as imposing nonnegativity on MK_i_ and MK_a_. Imposing signal monotonicity improved the precision only to a lesser extent (Figure 3).

Furthermore, even though the use of constraints can introduce a bias (Figure 3), the increase in precision and reduction in RMSE justify this minor loss in accuracy. When comparing CIWLLS3 to IWLLS using a lengthy 120‐sample acquisition scheme, the median relative increase in precision ranges from a factor of 1.08 for MK_a_, up to a factor of 1.38 for OP. The median reduction in RMSE of CIWLLS3 versus IWLLS ranges from a factor of 0.92 for MD, down to a factor of 0.71 for OP. The merits of using constrained estimators become even more evident in the case of limited data where the scalar DTD parameter maps are visibly less noisy when using the CIWLLS3 estimator instead of IWLLS (Figure 7).

### 5.4 Limitations

A general limitation of the QTI framework as proposed by Westin et al. (2016) is that it assumes that the system consists of multiple Gaussian diffusion components resulting in vanishing intra‐compartmental kurtosis, also known as microscopic kurtosis. As a result, it considers only two sources of kurtosis: isotropic kurtosis (arising from variance in isotropic diffusivities) and anisotropic kurtosis (arising from structural anisotropy). The correlation tensor imaging (CTI) framework (Henriques et al., 2020; Novello et al., 2022) can be seen as more general as it does not make this assumption and considers an additional source of kurtosis called microscopic kurtosis (arising from cross‐sectional variance, structural disorder, and restriction). As this article is built around the QTI technique, it does not consider the possibility of non‐zero microscopic kurtosis.

Moreover, under certain conditions, microscopic kurtosis can become negative. For example, in the event of edema with cell swelling, Alves et al. (2022) have shown in simulations that microscopic kurtosis can become negative. In a limited regime [see figure 2c of Alves et al. (2022)], this could result in the total isotropic kurtosis becoming negative, thus violating the non‐negativity constraint that we impose on isotropic kurtosis. More recently, CTI was deployed in the healthy human brain where the microscopic kurtosis was shown to be relatively small compared to MK_i_, but more relevantly, it appears to be exclusively positive (Novello et al., 2022). Thankfully, the constrained solvers proposed in this work can be trivially adapted to accommodate negative lower bounds on isotropic kurtosis.

We would like to point out that we used the CRLB to arrive at an optimized acquisition scheme by predicting the precision under ideal circumstances (e.g., normally distributed data, constant sigma, no artifacts other than noise, the model perfectly predicts the data, etc.). As is always the case with real data, there is a departure from these perfect assumptions. The data will not be normally distributed, the sigma will not be constant throughout the brain, there will be other artifacts than just noise and the model will not perfectly predict the data. Moreover, real data undergoes many preprocessing steps apart from just denoising, including Gibbs ringing correction and motion and eddy current distortion correction, all of which will introduce departures from the perfect assumptions. However, this does not preclude the CRLB‐optimized schemes from outperforming the reference schemes, even in real data where not all these assumptions are perfectly met.

## 6 CONCLUSION

Given the great interest in QTI and its ability to provide more specific tissue metrics than conventional DW MRI approaches, we investigated the precision and accuracy with which various scalar DTD parameters can be estimated. These depend both on the DW data acquisition scheme, as well as the estimator used.

We optimized QTI acquisition schemes for maximal precision of either the raw DTD tensor parameters or scalar DTD parameters. We obtained two parsimonious 8‐min acquisition schemes: one that provides a maximally precise estimation of the raw DTD parameters, and the recommended scheme that provides the maximally precise estimation of the scalar DTD parameters MD, FA, μFA, MK_i_, MK_a_, and OP.

Additionally, we found that using the iteratively weighted linear least squares estimator provides more accurate estimates compared to a weighted linear least squares estimator, and more precise estimates compared to a conventional linear least squares estimator. We also found that the use of constraints for both the scalar and tensor DTD parameters can significantly improve the precision of the iteratively weighted linear least squares estimator. As such, we hope to facilitate the adoption of QTI by both the clinic and the research community.

## CONFLICT OF INTEREST

Filip Szczepankiewicz is an inventor on patents related to gradient waveform design. Jan Morez, Arnold J. den Dekker and Jan Sijbers have no conflicts of interest to declare.

## Supporting information

Supporting Information

## Data Availability

The data that support the findings of this study are available from the corresponding author upon reasonable request. The free gradient waveform pulse sequence is available for multiple MRI systems and vendors, as described here: https://github.com/filip-szczepankiewicz/fwf_seq_resources. The acquisition schemes and constrained estimators will be freely available on GitHub.

## Footnotes

^1^
The benchmark estimator for the real data experiments was chosen based on the outcome of the simulation experiments (see Section 4.2). When choosing the benchmark estimator used for the real data experiments, we chose the estimator that performed best in the simulations (lowest bias and standard deviation), which was the IWLLS. Since the benchmark estimator also made use of the superset of data and on top of that used denoising, this way we obtained a reference with the best possible accuracy and precision.
