# Supplementary material for: Optimal experimental design and estimation for q‐space trajectory imaging
Source: Hum Brain Mapp. 2022 Dec 23;44(4):1793–809. doi: 10.1002/hbm.26175 (PMC9921251; doi:10.1002/hbm.26175)
Supplement: Supplementary file 1 — Supporting Information [file HBM-44-1793-s001.docx]

Supplemented material to

**Optimal experimental design and estimation for q- space trajectory imaging**

by

Jan Morez, Filip Szczepankiewicz, Arnold J. den Dekker, Floris Vanhevel, Jan Sijbers, Ben Jeurissen

Figure s1: The distribution of QTI samples of the optimized schemes as a function of b-value and b-tensor shapes, prior to clustering.


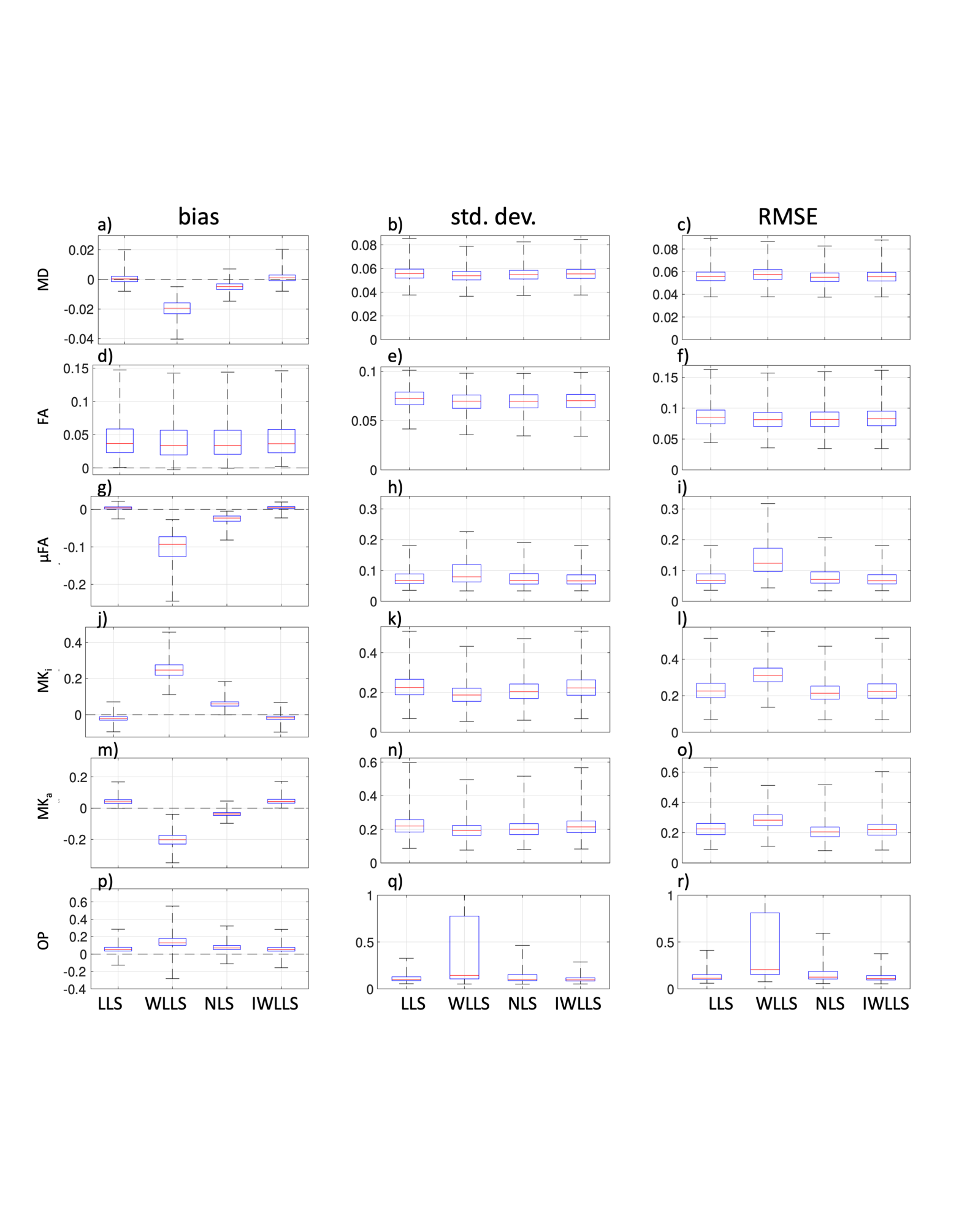


Figure s2: Simulations: bias, standard deviation, and RMSE of the different estimators for: MD (in $\mu m/ms^{2}$) (a-c), FA (d-f), $\mu FA$ (g-i), $MK_{a}$ (j-l), $MK_{i}$ (m-o), OP (p-r) of WM and GM voxels across the whole brain at SNR = 15. The distance between the whiskers is five times the interquartile width.

| Acq. scheme |  | Szczepankiewicz *et al.*, 2019 |  |
| --- | --- | --- | --- |
| b ($\mathrm{ms}/\mu m^{2})$ | PTE | STE | LTE |
| 0 | 4 | 5 | 4 |
| 0.1 | 10 | 50 | 10 |
| 0.7 | 10 | 50 | 10 |
| 1.4 | 16 | 50 | 16 |
| 2 | 46 | 50 | 46 |

Table s1: The acquisition scheme used by Szczepankiewicz et al. (2019).
